# Reassessing the Role of the Active TGF-*β*1 as a Biomarker in Systemic Sclerosis: Association of Serum Levels with Clinical Manifestations

**DOI:** 10.1155/2016/6064830

**Published:** 2016-11-14

**Authors:** Andréa Tavares Dantas, Sayonara Maria Calado Gonçalves, Anderson Rodrigues de Almeida, Rafaela Silva Guimarães Gonçalves, Maria Clara Pinheiro Duarte Sampaio, Kamila de Melo Vilar, Michelly Cristiny Pereira, Moacyr Jesus Barreto de Melo Rêgo, Ivan da Rocha Pitta, Claudia Diniz Lopes Marques, Angela Luzia Branco Pinto Duarte, Maira Galdino da Rocha Pitta

**Affiliations:** ^1^Hospital das Clínicas, Universidade Federal de Pernambuco (UFPE), Recife, PE, Brazil; ^2^Laboratório de Imunomodulação e Novas Abordagens Terapêuticas, Núcleo de Pesquisa em Inovação Terapêutica Suely Galdino, Recife, PE, Brazil; ^3^Laboratório de Planejamento e Síntese de Fármacos, UFPE, Recife, PE, Brazil

## Abstract

*Objective*. To determine active TGF-*β*1 (aTGF-*β*1) levels in serum, skin, and peripheral blood mononuclear cell (PBMC) culture supernatants and to understand their associations with clinical parameters in systemic sclerosis (SSc) patients.* Methods*. We evaluated serum samples from 56 SSc patients and 24 healthy controls (HC). In 20 SSc patients, we quantified spontaneous or anti-CD3/CD28 stimulated production of aTGF-*β*1 by PBMC. The aTGF-*β*1 levels were measured by ELISA. Skin biopsies were obtained from 13 SSc patients and six HC, and TGFB1 expression was analyzed by RT-PCR.* Results*. TGF-*β*1 serum levels were significantly higher in SSc patients than in HC (*p* < 0.0001). Patients with increased TGF-*β*1 serum levels were more likely to have diffuse subset (*p* = 0.02), digital ulcers (*p* = 0.02), lung fibrosis (*p* < 0.0001), positive antitopoisomerase I (*p* = 0.03), and higher modified Rodnan score (*p* = 0.046). Most of our culture supernatant samples had undetectable levels of TGF-*β*1. No significant difference in TGFB1 expression was observed in the SSc skin compared with HC skin.* Conclusion*. Raised active TGF-*β*1 serum levels and their association with clinical manifestations in scleroderma patients suggest that this cytokine could be a marker of fibrotic and vascular involvement in SSc.

## 1. Introduction

Since the discovery of the potent profibrotic and immunomodulatory activities of transforming growth factor-*β* (TGF-*β*), this cytokine has been considered a central player in the process of fibrogenesis in systemic sclerosis (SSc) and other fibrotic diseases. Its effects interfere with a wide array of cellular functions that are relevant to fibrosis, including differentiation of myofibroblasts, production of extracellular matrix molecules, and decreased synthesis of collagen degrading metalloproteinases. These activities lead to upregulation of collagen and extracellular matrix synthesis and, consequently, fibrosis [1, 2].

There are three isoforms of TGF-*β*, each encoded by a distinct gene, with a distinct expression pattern: TGF-*β*1, TGF-*β*2, and TGF-*β*3. Although increased expression of all the three isoforms has been observed in patients with SSc, TGF-*β*1 has been the most extensively studied. Secreted as a latent form, TGF-*β* has to be activated at the target site to induce cellular responses. Since activation of latent TGF-*β* is critical for the functioning of this growth factor, production levels of active TGF-*β* are likely to be more important than the levels of the latent form [3].

The blockade of TGF-*β* as a treatment strategy in SSc is currently under investigation, with conflicting results [4–6]. The important role of TGF-*β* in tissue fibrosis suggests that measurements of its serum levels may reflect the activity of the fibrotic process. However, there have been discordant reports about concentrations of TGF-*β* in the peripheral blood of patients with SSc [7–12].

Therefore, the current study was conducted to explore whether relationships exist between active TGF-*β*1 levels and clinically relevant parameters. The study analyzed serum, culture supernatants from peripheral blood mononuclear cells (PBMC), and skin from systemic sclerosis patients.

## 2. Methods

### 2.1. Study Subjects

Patients were recruited from the outpatient clinic at the Hospital das Clínicas of Universidade Federal de Pernambuco (UFPE), Brazil. We included 56 SSc patients (53 female patients, mean age 45.1 ± 14.3 years, and range 19–79) and 24 healthy controls (22 female volunteers, mean age 45.2 ± 11.8 years, and range 23–68). Patients fulfilled the preliminary criteria of the American College of Rheumatology (ACR) for the classification of SSc [13]. Patients with overlapping diseases were excluded. The study was approved by the ethics committee of the UFPE (CEP/CCS/UFPE 529/11) and, according to the Declaration of Helsinki, written informed consent was obtained from each subject before being included in the study.

SSc patients were classified into two categories: diffuse cutaneous systemic sclerosis (dSSc) and limited cutaneous systemic sclerosis (lSSc) [14]. Disease duration was defined as the time from onset of the first non-Raynaud manifestation of SSc. Clinical and laboratorial manifestations were reviewed from the medical charts. Lung fibrosis was observed using high-resolution computed tomography (HRCT); esophagus involvement was determined by scintillography and/or endoscopy; proximal muscle weakness and elevated serum creatine kinase indicated muscle involvement. Pulmonary artery pressure was estimated by Doppler echocardiogram; pressures above 35 mmHg were interpreted as pulmonary hypertension [15, 16]. The modified Rodnan total skin thickness score (mRSS) was used to evaluate skin fibrosis [17]. Clinical characteristics of the SSc patients are reported in Table 1.

### 2.2. PBMCs Purification and Culture

PBMCs were obtained from the heparinized blood samples of 20 SSc patients. The PBMCs were isolated using the standard Ficoll-Hypaque density-gradient centrifugation (GE Healthcare Biosciences, Pittsburgh, PA, USA) method. PBMCs (1 × 10^6^ cells/mL) were cultured in RPMI-1640 (Gibco) supplemented with 10% fetal bovine serum (Gibco, Carlsbad, CA, USA), HEPES 10 mM (Gibco, Carlsbad, CA, USA), and penicillin (10.000 U/mL)/streptomycin (10.000 *μ*g/mL) (Gibco, Carlsbad, CA, USA). Cells were stimulated with anti-CD3/CD28 (eBioscience, San Diego, CA, USA), and after 48 h culture supernatant was collected for quantification of cytokines.

### 2.3. Measurement of Serum TGF-*β*1 Levels

Fresh venous blood samples were centrifuged shortly after clot formation. All samples were stored at −70°C prior to use. Active TGF-*β*1 concentrations in serum and culture supernatants were determined by specific ELISA kit according to the manufacturer's recommendation (eBioscience, San Diego, CA, USA). Each sample was tested in duplicate. The detection limit of the test according to the manufacturer was 8 pg./mL and the standard curve covered a concentration range from 8 to 1000 pg./mL.

### 2.4. RNA Isolation and Real-Time Polymerase Chain Reaction (RT-PCR)

Skin biopsies were obtained from 13 SSc patients. Skin samples of five healthy controls were obtained by plastic surgery from individuals without any other autoimmune diseases. All biopsies were taken from a lesional forearm site with a punch of 5 mm. Samples were stored in RNAlater (Qiagen, Hilden, Germany). Tissues were homogenized using* TissueRuptor *(QIAGEN). Total RNA was isolated from tissues with RNeasy Mini Kit (QIAGEN Sciences, Maryland, USA) or TRIzol (Invitrogen), in both cases according to the manufacturers' instructions. For cDNA synthesis, 4–6 *μ*g of total RNA was used.* High-Capacity cDNA Reverse Transcription Kit *(Applied Biosystems, Warrington, UK) was used according to manufacturer's protocol. TGFB1 mRNA levels were measured by quantitative real-time Polymerase Chain Reaction (qRT-PCR) using 18S ribosomal gene as the internal standard. Standard TaqMan probe was Hs00998133_m1 for TGFB1. Real-time PCR reactions were performed on ABIPrism 7900HT sequence detection PCR machine (Applied Biosystems) according to the manufacturer's protocol. Data were analyzed with SDS version 2.3 software using the comparative Ct  (2^−ΔΔCt^) method.

### 2.5. Statistical Analysis

Statistical analyses of the data were performed using the GraphPad Prism 6.0 (GraphPad Software Inc., San Diego, CA) statistical program. D'Agostino test verified the normality of samples. Numerical data were expressed as mean ± standard deviation (SD) or median and interquartile range (IQR) according to their distribution. The Mann–Whitney* U* test was used to compare serum cytokines levels. Spearman's rank correlation coefficient was used to examine the relationship between two continuous variables. We considered correlation (*R* ^2^) strength as follows: 0 < *R* ^2^ ≤ 0.35 = weak correlation; 0.35 < *R* ^2^ ≤ 0.67 = moderate correlation; 0.67 < *R* ^2^ ≤ 1 = strong correlation. The association between TGF-*β*1 levels and the different internal organs involved was studied by univariate and multivariate analyses. In univariate analysis, we used the above-mentioned test (Mann–Whitney* U* test) to compare variables in subjects with or without organ involvement. To evaluate the association between the organs involved and different biological variables that were present after simultaneously adjusting the other variables of interest, we performed multiple linear regression analysis for the clinical variables with linear scores and multiple logistic regression for the clinical variables with dichotomous scores. A probability value of* p* < 0.05 was considered significant.

## 3. Results

### 3.1. Serum TGF-*β*1 Levels in SSc Patients

**Figure 1:**
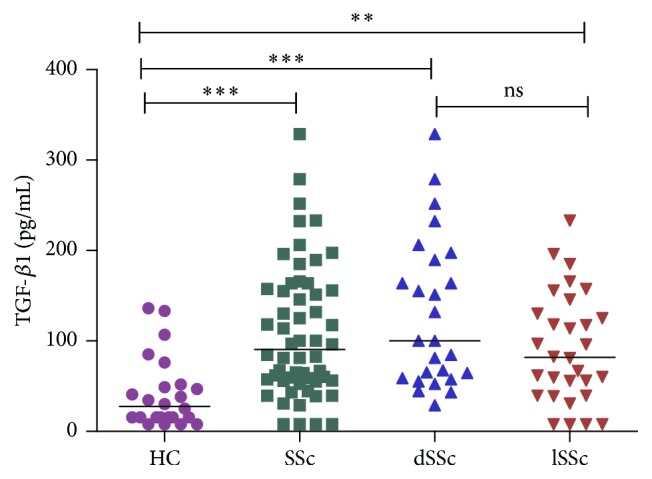
Serum levels of active TGF-*β*1 in healthy controls (HC), systemic sclerosis (SSc) patients, diffuse cutaneous systemic sclerosis (dSSc) patients, and limited cutaneous systemic sclerosis (lSSc) patients. Short horizontal bars represent median.  ^*∗∗*^ *p* = 0.001,  ^*∗∗∗*^ *p* < 0.0001, and ns = not significant.

TGF-*β*1 serum levels were significantly higher in SSc patients [90.38 (55.84–157.1) pg./mL] than in healthy control subjects [27.56 (15.63–51.16) pg./mL,* p* < 0.0001)]. When SSc patients were classified into cases of lSSc and dSSc, both lSSc patients [81.94 (39.44–134.0)] and dSSc [(100.1 (58.50–191.5)] patients had significantly higher TGF-*β*1 levels compared to healthy controls (*p* = 0.001 and* p* < 0.0001, resp.). Among the SSc subsets, there was no significant difference in serum TGF-*β* 1 levels between dSSc and lSSc patients (Figure 1).

### 3.2. Increased Serum TGF-*β*1 Levels Are Associated with Clinical Manifestations

In order to evaluate the association between serum levels and clinical manifestations, we classified the patients based on the presence/absence of the main clinical parameters. In a univariate analysis, we found that serum TGF-*β*1 levels were higher in SSc patients with lung fibrosis (*p* = 0.004), digital ulcer (*p* = 0.02), and positive antitopoisomerase I (*p* = 0.04) compared to patients without these manifestations (Table 2).

To confirm these associations, these variables were further subjected to multivariate logistic regression analysis adjusted for age, gender, disease duration, and treatment. In this analysis, lung fibrosis and positive antitopoisomerase I were independently associated with TGF-*β*1 levels (*p* < 0.0001 and* p* = 0.03, resp.). Also, patients with higher circulating TGF-*β*1 levels were more likely to have diffuse cutaneous form (*p* = 0.02) (Table 2). Finally, we performed a logistic regression analysis for continuous variables also adjusted for age, gender, disease duration, and treatment. By this analysis we found that TGF-*β*1 levels were associated with modified Rodnan score (regression coefficient = 0.03,* p* = 0.046).

### 3.3. mRNA Expression of TGFB1 in Skin Biopsies

Next, we evaluated TGFB1 mRNA expression in skin biopsies of 13 SSc patients and six healthy controls. TGFB1 mRNA was expressed in all SSc skin biopsies but only in four healthy control biopsy samples. No significant difference in TGFB1 expression was observed in the SSc skin compared with HC (relative mRNA levels 1.3 ± 0.3 and 1.2 ± 0.8,* p* = 0.24). There were no differences in TGFB1 skin expression between those with lSSc and those with dSSc (data not shown). No significant association was observed between mRNA levels and clinical manifestations (data not shown).

### 3.4. TGF-*β*1 Levels in PBMC Cultures

We next addressed spontaneous and stimulated (anti-CD3/CD28) TGF-*β*1 production by SSc PBMCs. However, among 20 SSc patients, only six presented detectable levels of active TGF-*β*1 in supernatants of stimulated and/or nonstimulated PBMCs cultures.

## 4. Discussion

We used different biological sources to assess the role of active TGF-*β*1 as a biomarker in SSc. Although it is still a controversial issue in the literature, some studies have indirectly suggested the potential usefulness of TGF-*β*1 levels as a biomarker. This came to light when they evaluated the reduction of TGF-*β*1 after specific treatments [18, 19]. Most of the studies have failed to establish a relationship between TGF-*β*1 serum levels and clinical features in SSc patients [7–9] but, in our study, we demonstrated a significant association between higher serum TGF-*β*1 levels and diffuse subset, digital ulcers, lung fibrosis, skin involvement, and positive antitopoisomerase I.

Previous studies have reported conflicting results on the utility of serum TGF-*β*1 [7–12]. We observed significantly higher serum TGF-*β*1 levels in SSc patients. Our findings are in agreement with what had been suggested by other authors [7, 11, 12]. However, some studies found no differences in TGF-*β*1 levels between SSc patients and controls [8, 9] or even lower categories [10]. These studies could have posted different results on account of differences in the study population (disease duration, treatment), techniques used, and the molecules evaluated (total or active TGF-*β*1).

Although the univariate analysis has not identified differences in mean TGF-*β*1 serum levels between dSSc and lSSc subsets, after adjustment for age, gender, disease duration, and treatment used, we found that patients with diffuse form were more likely to have higher active TGF-*β*1 levels. We also verified a positive correlation between TGF-*β*1 levels and the extent of skin fibrosis, as assessed by mRSS. Sato et al. [8] found that serum TGF-*β*1 levels tended to be higher in patients with dSSc than in patients with lSSc but there was no significant correlation with mRSS. An opposite result was described by Dziadzio et al. [10], where they demonstrated reduced levels of active TGF-*β*1 in dSSc and an inverse correlation with extent of skin fibrosis. This finding suggests that active TGF-*β*1 may be sequestered in involved SSc tissue. An important difference between this and our study was in the disease duration, since patients with established disease tend to have higher TGF-*β*1 levels compared with those in the early stages of disease [12]. While in the study by Dziadzio et al. patients with diffuse form had early disease (median 5 yrs.), our patients were in the later stages of disease (median 10 yrs.).

Despite the recognized participation of TGF-*β*1 in the development of pulmonary fibrosis, its role as biomarker of lung disease is undefined [20] as yet. Previous studies have identified increased TGF-*β*1 serum levels in patients with idiopathic pulmonary fibrosis [21, 22], but no previous association was observed in SSc patients. Furthermore, the data on the presence of increased expression of TGF-*β* in lung tissues of patients with SSc [23–25] are inconclusive. Our data showed that TGF-*β*1 levels were significantly higher in patients with lung fibrosis compared to healthy controls.

Corroborating our finding of an association between TGF-*β*1 levels and the diffuse cutaneous subset and pulmonary fibrosis, we observed higher TGF-*β*1 levels in patients with positive antitopoisomerase I. Furthermore, the presence of scleroderma-associated autoantibodies is correlated with distinct clinical phenotypes of SSc. Antitopoisomerase I antibodies are also associated with diffuse skin involvement and lung fibrosis and are correlated with a poor prognosis and SSc-related mortality [26].

We found higher TGF-*β*1 levels in patients with digital ulcers. Although TGF-*β* has been traditionally considered an important mediator of fibrosis, it can also contribute to vascular abnormalities in SSc. It was demonstrated that TGF-*β* induces the synthesis of endothelin, a potent vasoconstrictor implicated in ulcer pathogenesis in systemic sclerosis [27]. It was found that TGF-*β* acts synergistically with endothelin [28]. The vascular effect of TGF-*β*, through its interaction with the coreceptor endoglin, also results in activation of endothelial cells and vascular smooth muscle cells [29].

In order to better evaluate the association between clinical manifestations and TGF-*β*1 levels, we tried to measure TGF-*β*1 in other biological sources. Given that TGF-*β*1 is secreted by numerous cell types, including monocytes/macrophages and lymphocytes [30], we decided to investigate TGF-*β*1 spontaneous and stimulated production by PBMCs from SSc patients. Two previous studies had reported conflicting results on this line of investigation. Giacomelli et al. [31] demonstrated that total TGF-*β*1 production by PBMC is normal in SSc. Hasegawa et al. [32] showed increased spontaneous production of active TGF-*β*1 by PBMC from both lSSc and dSSc patients compared with controls. In this study, monocytes/macrophages were the main producing cells since secretion of TGF-*β*1 by T cells, B cells, and NK cells was undetectable [32]. Most of our samples were below the kit detection limit. As our patients are undergoing treatment with immunosuppressive and/or corticosteroids, it is possible that this may have affected the measurement. Furthermore, as the main cellular sources of TGF-*β*1 in peripheral blood are monocytes/macrophages, using a specific stimulus for T lymphocytes (anti-CD3/CD28) has not enabled increased production of TGF-*β*1.

We also could not demonstrate differences or clinical correlations in TGFB1 mRNA expression between skin biopsies of SSc patients and healthy controls. Matsushita et al. [11] evaluated only diffuse SSc patients and showed enhanced skin TGFB1 expression in SSc patients with early disease. Probably, our small patients' number was not enough to detect these differences.

Taken together, our findings demonstrated raised active TGF-*β*1 serum levels in SSc patients. Furthermore, we showed a significant association between active TGF-*β*1 serum levels and vascular (digital ulcers) and fibrotic (lung and skin) manifestations in SSc patients, suggesting that raised levels could be evaluated as a marker of advanced disease.

## Figures and Tables

**Table 1:** Demographic and clinical characteristics of the patients with systemic sclerosis (*n* = 56).

Characteristic	
*Age (yrs.), mean ± SD (range)*	45.1 ± 14.3 (19–79)
*Gender female, n (%)*	53 (94.6)
*Disease duration (months), median (range)*	120 (8–696)
*Clinical subgroups, n (%)*	
Diffuse cutaneous	26 (46.4)
Limited cutaneous	30 (53.6)
*Rodnan score, median (range)*	7.0 (0–36)
*Clinical manifestations, n (%)*	
Raynaud phenomenon	54 (96.4)
Digital ulcer	33 (58.9)
Esophageal dysfunction (*n* = 48)	28 (58.3)
Lung fibrosis	24 (42.9)
Pulmonary arterial hypertension (*n* = 54)	9 (16.7)
Arthritis	18 (32.1)
Muscle involvement	14 (25.0)
*Autoantibodies, n (%)*	
Positive ANA (*n* = 49)	46 (93.9)
Positive anticentromere (*n* = 44)	7 (15.9)
Positive antitopoisomerase I (*n* = 33)	14 (42.4)
*Treatment, n (%)*	
Glucocorticoids	20 (35.7)
Azathioprine	10 (17.9)
Methotrexate	4 (7.1)
Mycophenolate mofetil	3 (5.6)
Cyclophosphamide	6 (10.7)

**Table 2:** Associations of TGF-*β*1 levels with organ involvement in systemic sclerosis (*n* = 56).

Organ involvement	Status	TGF-*β*1 levels (pg/mL)	*p* value			
		Median [IQR]	Unpaired *t-*test	Model 1	Model 2	Model 3
Diffuse cutaneous subset	+	100.1 [58.5–191.5]	0.10	0.01	0.02	0.02
	−	81.9 [39.4–134.0]				
Digital ulcer	+	113.8 [66.0–187.3]	0.02	NS	NS	NS
	−	60.1 [44.4–125.1]				
Esophageal dysfunction	+	111.0 [55.4–161.8]	0.63	NS	NS	NS
	−	82.9 [59.0–118.0]				
Lung fibrosis	+	153.2 [81.3–180.2]	0.004	0.005	0.004	<0.0001
	−	64.7 [43.5–116.6]				
Pulmonary arterial hypertension	+	81.3 [55.4–134.8]	0.99	NS	NS	NS
	−	96.3 [55.4–163.8]				
Muscle involvement	+	106.9 [60.8–159.1]	0.48	NS	NS	NS
	−	82.9 [44.1–157.7]				
Arthritis	+	81.3 [43.2–151.1]	0.40	NS	NS	NS
	−	96.6 [58.5–163.0]				
Antitopoisomerase I	+	100.1 [67.6–232.6]	0.04	0.03	NS	0.03
	−	57.6 [34.7–131.6]				

Model 1 = adjusted for age and gender; Model 2 = adjusted for age, gender, and disease duration; and Model 3 = adjusted for age, gender, disease duration, and treatment. NS = not significant.
